# Integrated investigation of DNA methylation, gene expression and immune cell population revealed immune cell infiltration associated with atherosclerotic plaque formation

**DOI:** 10.1186/s12920-022-01259-z

**Published:** 2022-05-09

**Authors:** Yihong Yin, Zhaohong Xie, Dong Chen, Hao Guo, Min Han, Zhengyu Zhu, Jianzhong Bi

**Affiliations:** 1 Department of Neural Medicine, The Second Hospital of Shandong University, Shandong University, No. 247 Beiyuan Street, Jinan, 250033 China; 2 Department of Neurology, The First Affiliated Hospital of USTC, Division of Life Sciences and Medicine, University of Science and Technology of China, 17 Lujiang Road, Hefei, 230001 China; 3 Center for Genome Analysis, Wuhan Ruixing Biotechnology Co. Ltd, Wuhan, 430075 China

**Keywords:** Atherosclerosis, DNA methylation, Gene expression, Plaque, Immune cells

## Abstract

**Background:**

The clinical consequences of atherosclerosis are significant source of morbidity and mortality throughout the world, while the molecular mechanisms of the pathogenesis of atherosclerosis are largely unknown.

**Methods:**

In this study, we integrated the DNA methylation and gene expression data in atherosclerotic plaque samples to decipher the underlying association between epigenetic and transcriptional regulation. Immune cell classification was performed on the basis of the expression pattern of detected genes. Finally, we selected ten genes with dysregulated methylation and expression levels for RT-qPCR validation.

**Results:**

Global DNA methylation profile showed obvious changes between normal aortic and atherosclerotic lesion tissues. We found that differentially methylated genes (DMGs) and differentially expressed genes (DEGs) were highly associated with atherosclerosis by being enriched in atherosclerotic plaque formation-related pathways, including cell adhesion and extracellular matrix organization. Immune cell fraction analysis revealed that a large number of immune cells, especially macrophages, activated mast cells, NK cells, and Tfh cells, were specifically enriched in the plaque. DEGs associated with immune cell fraction change showed that they were mainly related to the level of macrophages, monocytes, resting NK cells, activated CD4 memory T cells, and gamma delta T cells. These genes were highly enriched in multiple pathways of atherosclerotic plaque formation, including blood vessel remodeling, collagen fiber organization, cell adhesion, collagen catalogic process, extractable matrix assembly, and platelet activation. We also validated the expression alteration of ten genes associated with infiltrating immune cells in atherosclerosis.

**Conclusions:**

In conclusion, these findings provide new evidence for understanding the mechanisms of atherosclerotic plaque formation, and provide a new and valuable research direction based on immune cell infiltration.

**Supplementary Information:**

The online version contains supplementary material available at 10.1186/s12920-022-01259-z.

## Introduction

Cardiovascular diseases are the most important threat tightly associated with life quality and health condition of all humans worldwide [1]. In most cases, the underlying cause of cardiovascular diseases is atherosclerosis, treated as the pathological basis of other cardiovascular diseases, including atherosclerotic cerebral infarction [2]. The pathogenesis of atherosclerosis is associated with a complex interplay of endothelial dysfunction [3], lipid accumulation [4], inflammation [5], vascular smooth muscle cell proliferation [6], matrix turnover, calcification [7], and other complex interactions representing the dynamic process from fat streaks to stable or unstable atherosclerotic plaques [8]. A cellular biology study demonstrated that atherogenic processes in multiple cell types were activated to induce atherosclerosis [9]. One of the key causes of atherosclerosis is the dysregulation of immune response and inflammation in the artery wall with the activation of T helper cells [10, 11]. Extensively understanding the underlying mechanisms could greatly help researchers and medical staff overcome atherosclerosis.

In the process of atherosclerosis, inflammatory response is accompanied by the increase of many proinflammatory factors, including MCP1, interferon-gamma (IFN-γ), IL-8, VCAM1 and TNF [10, 12]. Among them, oxidative low-density lipoprotein (ox LDL)-induced monocyte/macrophage inflammatory response is a key event in the pathogenesis of atherosclerosis [13, 14]. As an important factor is apolipoprotein E (ApoE) that could be treated as a therapeutic target by promoting clearance of lipoproteins and normalization of serum cholesterol levels in mice [15]. ApoE deficiency can lead to the accumulation of sphingomyelin-rich residues and induce macrophages to accumulate more cholesterol [16]. Recent studies have reported the relationship between abnormal DNA methylation and atherosclerosis [17, 18], and found that promoter methylation of ApoE and miRNA-223 genes are significantly associated with atherosclerotic cerebral infarction (ACI) [19, 20], indicating that epigenetic regulation affected by environment plays an important role in the pathogenesis of ACI. In atherosclerosis, macrophages and monocytes are exposed to inflammatory cytokines, oxidized lipids, cholesterol, and other factors. These factors could cause specific transcription reactions and interact with each other, resulting in transcriptional and apparent heterogeneity of macrophages in plaques [21].

Some innate immune cells play important roles in different stages of atherosclerotic development, but macrophages are the main type of innate immune effector cells in plaques. T cells are involved in the regulation of the plaque development [10]. The process of atherosclerosis is accompanied by significant changes of the immune cell infiltration [22]. In the early stage of atherosclerosis, macrophages, T cells and dendritic cells are recruited into the adventitia and surrounding vascular system [23]; in the late stage, the inflammation of adipose tissue will continue to increase, and the content of macrophages and B cells will also further increase [24]. Blood DNA methylation biomarkers have important application value in diagnosis, prediction, prognosis and treatment. In chronic inflammatory diseases, methylation module represents an immune component, and its specific performance is related to the changes in immune cell infiltration and distribution. Immunomethylation markers can be used as biomarkers of such diseases [25].

To further study the transcription outcome of DNA methylation influence, we performed an integration analysis based on the previously reported differential DNA methylation gene data between carotid atherosclerotic plaque and normal artery (GSE46401) in patients with atherosclerosis [26], and the differential expression gene data between carotid atherosclerotic plaque and peripheral blood monocytes (PBMCs) (GSE21545) [27]. We analyzed the abnormal gene expression level and DNA methylation (DNAm) level in atherosclerotic plaque or PBMC samples, then we validated the expression changes using the RT-qPCR experiment. Finally, we further studied the correlation between DNA methylation-related differentially expressed genes and different cell type changes, which could provide a potential link between DNA methylation, gene expression, and cell types in atherosclerotic plaque.

## Materials and methods

### DNA methylation (DNAm) analysis

DNA methylation microarray data was downloaded from the NCBI GEO database (GSE46401) [26]. A high-density (485,577 CpG sites) DNA methylation microarray (Infinium HumanMethylation450 BeadChip) was utilized to identify specific loci of differential DNA methylation with a set of donor-matched aortic samples, including 19 stable and advanced atherosclerotic carotid samples (carotid), 15 atherosclerotic lesion samples (A), and 15 matched normal aortic tissue samples (N). Quality control, data normalization, and statistical filtering procedures were performed according to the published paper [26]. The methylation levels of detected probes that were associated with genes were used to perform differential methylation statistical analysis between 15A and 15 N samples (paired *t*-test, Bonferroni-corrected *p* value < 1 × 10^–7^). Genes with differentially methylated probes were used to perform functional enrichment analysis.

### Transcriptome analysis

In this project, we downloaded the transcriptome microarray datasets GSE43292 (34 atheroma plaques (ATH) and 34 macroscopically intact tissue) [28] and GSE21545 (126 carotid plaques in patients with atherosclerosis *vs.* 98 peripheral blood mononuclear cell (PBMC) samples, including 97 paired samples) using Affymetrix HG-U133 plus 2.0 oligonucleotide arrays [27]. Gene expression profile was obtained from the Gene Expression Omnibus database (https://www.ncbi.nlm.nih.gov/geo). Raw data processing, quality control, data normalization and filtering were done according to the published paper [27]. The microarray probes were transformed into gene symbols according to annotation. If several probes were mapped to one gene symbol, the mean density of these probes was set as the final expression value of this gene. We also used limma package [29] to consider the age covariate (detailed gender of each sample was not provided in the published paper). We found that the differentially expressed genes were the same, indicating the small contribution of age covariate. Thus, we used online GEO2R with default parameters (https://www.ncbi.nlm.nih.gov/geo/geo2r/) to compare the two groups in order to identify genes that were differentially expressed under experimental conditions. Two thresholds, including adjusted *p* value < 0.05 and |log2fold change (FC)|> = 1, were set as the cut-off criteria. We then analyzed the differentially expressed genes (DEGs) by principal component analysis and functional enrichment analysis.

In this project, we analyzed the association between DEGs and DMGs obtained from the two studies, and recognized the gene expression changes related to DNA methylation. DEGs and DMGs were overlapped to identify the co-regulated genes at both DNA methylation and transcriptional levels. The DEGs were classified into two classes: DNA methylated (with DMG) and DNA non-methylated (without DMG).

### Cell-type quantification

Atherosclerosis is a chronic inflammatory disease with dysregulated fractions and functions of immune cells [10], so it is important to decipher the fraction changes of immune cells in carotid plaques versus normal samples. Based on all detected genes from GSE21545 transcriptome microarray data, the types of immune cells in each sample group were analyzed. An R package, immunedeconv [30] that provides a unified interface to seven deconvolution methods, was used for estimating immune cell fractions. Besides, CIBERSORT method [31] was applied in this study. The CIBERSORT algorithm is the most widely used deconvolution method, which characterizes its cell composition from the gene expression profile of complex tissues. Its results have been shown to correlate well with flow cytometric analysis. We also tested other two software, including immunecellAI [32] and EPIC [33], but they showed cell fraction bias or less cell types compared with CIBERSORT. With the default parameter, CIBERSORT was finally adopted to estimate immune cell fractions using expression values of all expressed genes. A total of 22 human immune cell phenotypes can be deconstructed by CIBERSORT, including 7 T cell types [CD8 T cells, naïve CD4 T cells, memory CD4 resting T cells, memory CD4 activated T cells, T follicular helper cells, and regulatory T cells (Tregs)]; naïve and memory B cells; plasma cells; resting and activated NK cells; monocytes; macrophages M0, M1, and M2; resting and activated dendritic cells; resting and activated mast cells; eosinophils; and neutrophils.

### RT-qPCR experiment

To further validate the immune cell type changes which could be reflected by the marker gene expression changes, we performed an RT-qPCR experiment to explore the deregulated gene expression levels. We extracted PBMCs from 15 atherosclerosis and 15 normal samples from The First Affiliated Hospital of University of Science and Technology of China and tested the expression levels of 10 selected genes. This process was approved by the ethics committee of First Affiliated Hospital of University of Science and Technology of China (2021KY131), and all volunteers. Informed consent was obtained from all subjects and/or their legal guardian(s). All methods were employed in accordance with the relevant guidelines and regulations. Clinical information of these patients and volunteers was provided in Additional file 2: Table S1. We have strictly followed the standard biosecurity and institutional safety procedures in our country (Biosecurity Law of People’s Republic of China). All the blood samples were processed immediately after collection for the isolation of peripheral blood mononuclear cells (PBMCs). The PBMCs were extracted according to the previously described method [34], and then stored at − 80 °C before RNA extraction.

First, total RNAs were extracted from PBMCs using TRIzol reagent (Invitrogen) according to the manufacturer’s instructions. The RNA integrity of each sample was estimated using a 1.5% agarose gel electrophoresis and quantified by spectrometer. Then, 10 μg of the purified RNA was reverse-transcribed and taken for complementary DNA with PrimeScript RT reagent Kit (Takara). Subsequently, qRT-PCR was conducted using TB Green Fast qPCR Mix (Takara) and specific primers (Additional file 3: Table S2) under the following amplification conditions: denaturing at 95 °C for 30 s, followed by 40 cycles of denaturing at 95 °C for 10 s and annealing and extension at 60 °C for 30 s. Relative gene expression was determined by employing the 2^−ΔΔCT^ method and normalized against U6 RNA. Mann–Whitney U test was carried out to determine the expression differences between sepsis and control groups. Statistical analyses were carried out using GraphPad Prism software [35]. All *P* values are two-sided. *P* < 0.05 was considered statistically significant.

### Functional enrichment analysis

Gene Ontology (GO) terms and Kyoto Encyclopedia of Genes and Genomes (KEGG) pathways were identified using the KOBAS 2.0 server to investigate the comprehensive set of functional annotations of a large list of genes. The Benjamini–Hochberg FDR controlling procedure and the hypergeometric test were used to define the enrichment of each term. Reactome pathway profiling (http://reactome.org) was also used for the functional enrichment analysis of the sets of selected genes. A *p* value < 0.005 was set as the cutoff criterion.

### Other statistical analysis

Principal component analysis (PCA) was performed with R package factoextra (https://cloud.r-project.org/package=factoextra) to show the clustering of samples with the first two components for both DNA methylation and transcriptome microarray datasets. After normalizing the density values of each gene/probe in samples, an in-house script (sogen) was used for visualization of next-generation sequence data and genomic annotations. The pheatmap package (https://cran.r-project.org/web/packages/pheatmap/index.html) in R was used to perform the clustering based on Euclidean distance. Student’s *t*-test was used for comparisons between two groups.

## Results

### Analysis of the hypermethylated genes previously identified in atherosclerotic aortas and carotid plaques

**Fig. 1:**
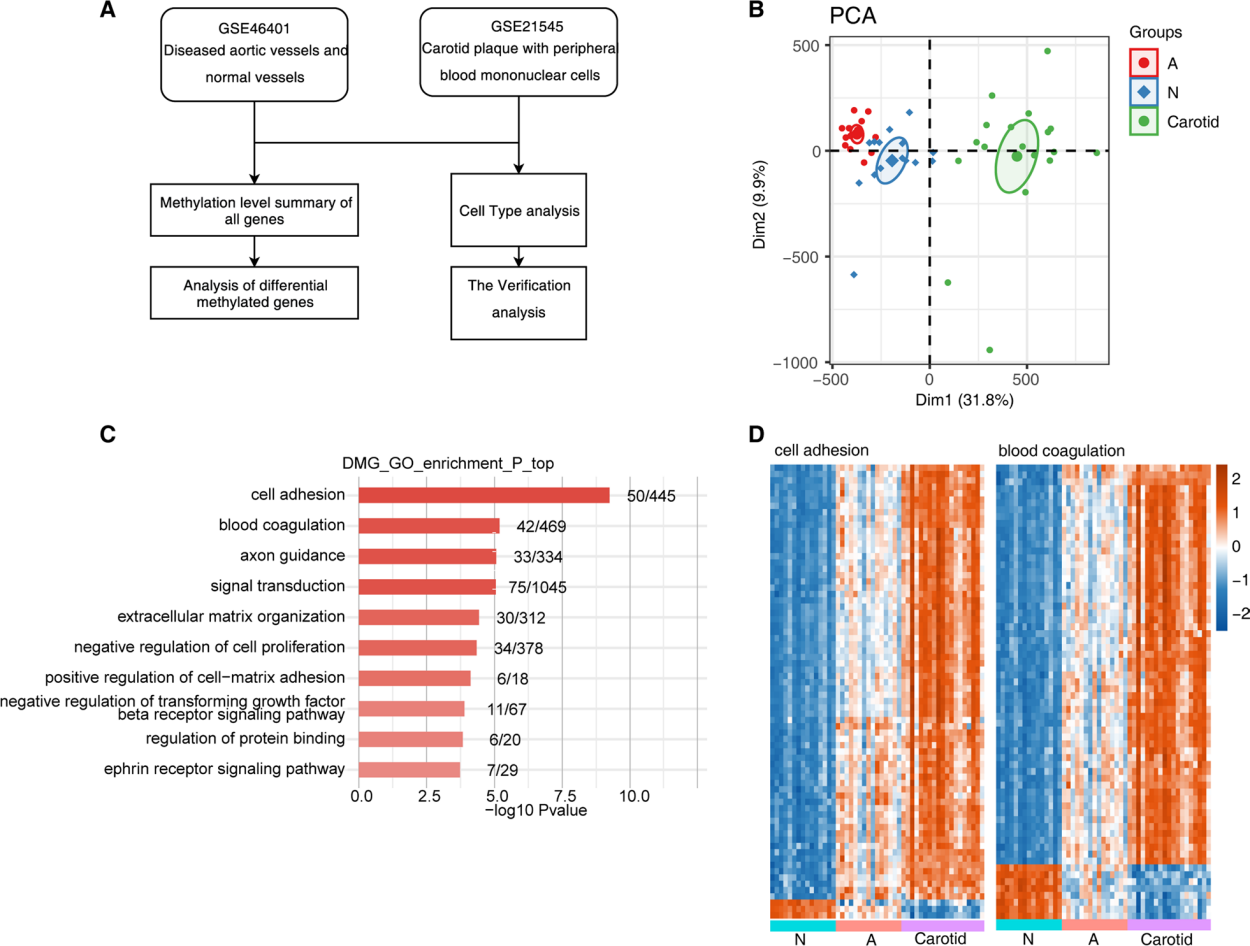
Analysis of the hypermethylated genes previously identified in atherosclerotic aortas and carotid plaques. (**A**) Workflow diagram showing the exploration pipeline. (**B**) Principal component analysis (PCA) of atherosclerotic plaque vs non-atherosclerotic aortic vascular samples based on DNA methylation level. The samples were grouped by disease state and the ellipse for each group is the confidence ellipse. Confidence interval is 0.95. (**C**) Bar plot showing the top 10 most enriched GO terms of differential methylated genes (DMGs). (**D**) Heatmap showing the DNA methylation level of DMGs involved in cell adhesion and blood coagulation

To further interpret the underlying molecular mechanisms in atherosclerosis, we downloaded the DNA methylation microarray data associated with atherosclerosis [26], containing 19 stable and advanced atherosclerotic carotid samples (carotid), 15 atherosclerotic lesion samples (A), and 15 matched normal aortic tissue samples (N). In the referred study, the 19 stable and advanced atherosclerotic carotid samples were used to validate the differentially methylated CpGs (dmCpGs) that don’t have regional epigenetic changes or batch effects; the results showed a very high consistency (98% of dmCpGs) [26]. Thus, we included the 19 carotid samples in our analysis to analyze the methylation levels of dmCpGs identified from the 15 paired samples. We then figured out the differentially methylated genes (DMGs) between the 15A samples and 15 N samples (Fig. 1A, Additional file 4: Table S3). After obtaining the DMGs, we performed principal component analysis (PCA) to explore the methylation pattern among the three groups (Fig. 1B). The top two components could explain 41.7% of the total variation, and the first component explained 31.8%. The three groups could be separated by the first component (Fig. 1B), suggesting the obvious differential methylation among these three groups. We then performed functional enrichment analysis for these DMGs. Gene ontology (GO) analysis revealed that the top ten enriched biological processes (BPs) included cell adhesion, blood coagulation, axon guidance, signal transduction, and extracellular matrix organization (Fig. 1C). We extracted the detailed methylation levels of genes from cell adhesion and blood coagulation pathways. Most of these genes showed increased methylation level in carotid samples, and the methylation levels of these DMGs showed a gradual increase or decrease from normal to advanced atherosclerotic development (Fig. 1D). KEGG pathway analysis also demonstrated that the focal adhesion and ECM-receptor interaction pathways were enriched with top *p*-value (Additional file 1: Fig. S1A). Reactome analysis was carried out to further explore the DMG functions. Translocation of ZAP−70 to immunological synapse, phosphorylation of CD3 and TCR zeta chains, and PD−1 signaling, which were related to immune response, were the top three enriched pathways (Additional file 1: Fig. S1B). These results suggest that ECM and immune response-related pathways may be related to the changes of collagen fibrin in carotid atherosclerotic plaque.

### Transcriptome analysis of deregulated gene expression in atherosclerotic carotid plaques

**Fig. 2:**
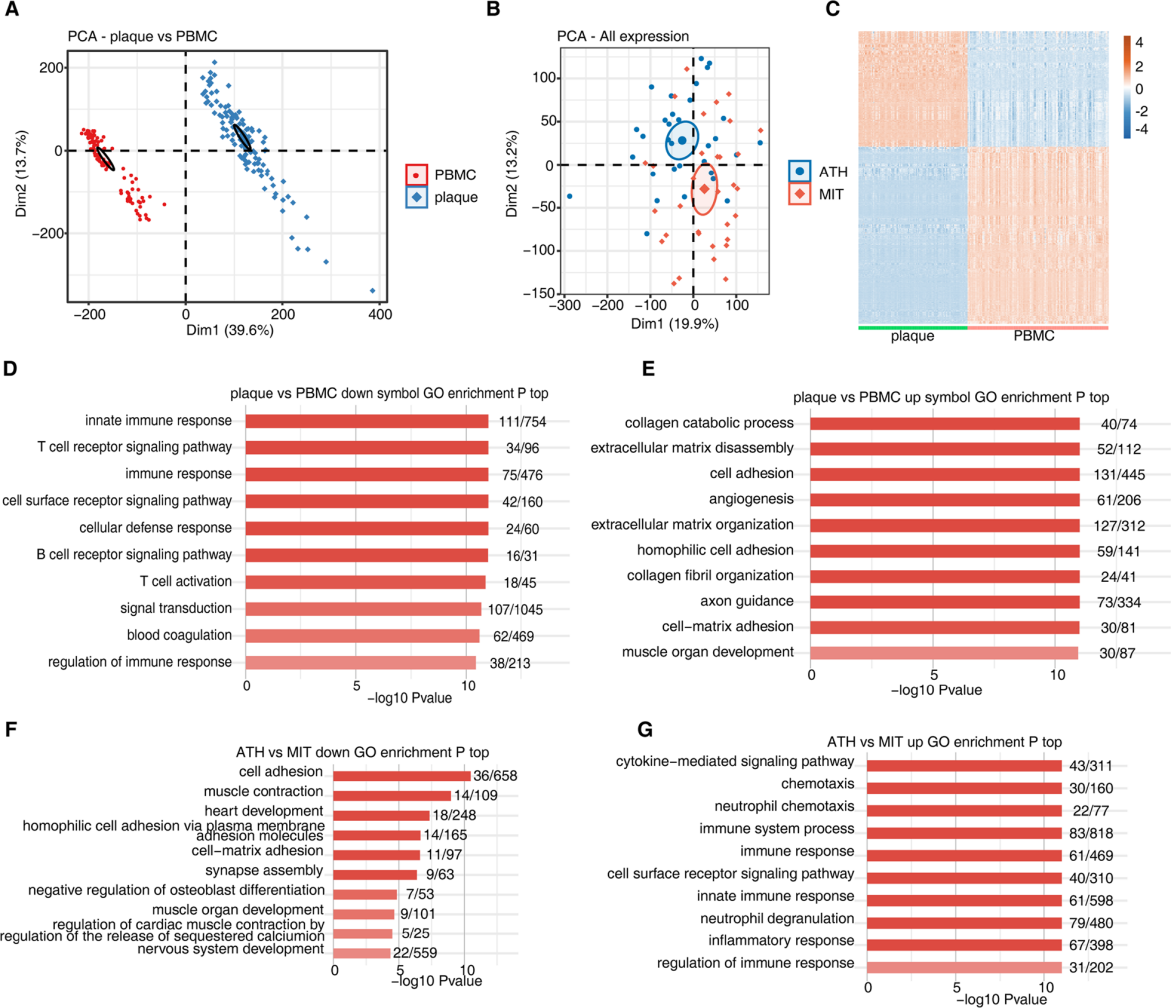
Transcriptome analysis of deregulated gene expression in atherosclerotic carotid plaques. (**A**) Principal component analysis (PCA) of atherosclerotic carotid plaque *vs.* PBMCs samples based on gene expression level. The samples were grouped by disease state. The ellipse for each group is the confidence ellipse. Confidence interval is 0.95. (**B**) The same with A but for ATH *vs.* MIT samples. (**C**) Expression heatmap of all differentially expressed genes between plaque *vs.* PBMCs. (**D**–**E**) The top 10 most enriched GO terms of down-regulated (**D**) and up-regulated (**E**) genes from differential expressed genes between plaque *vs.* PBMCs. (**F**–**G**). The top 10 most enriched GO terms of up-regulated (**F**) and down-regulated (**G**) genes from differential expressed genes between ATH *vs.* MIT

The DNA methylation level of CpG islands at the promoter region of genes was tightly associated with their transcriptional level. To uncover how the DMGs were expressed between atherosclerotic carotid plaques and normal samples, we downloaded two expression profiling datasets, including GSE43292 (34 atheroma plaque (ATH) and 34 macroscopically intact tissue (MIT)) [28] and GSE21545 (126 carotid plaques in patients with atherosclerosis *vs.* 98 peripheral blood mononuclear cell (PBMC) samples, including 97 paired samples) [27]. After normalizing the expression level, PCA result showed that the plaque samples were clearly separated from PBMC samples by the first component (Fig. 2A), while the ATH and MIT samples were not clearly separated (Fig. 2B). We then performed differentially expressed genes (DEGs) analysis for these two datasets. We finally obtained 1551 up DEGs and 1158 DEGs in plaque *vs.* PBMCs pair, as well as 512 up DEGs and 358 down DEGs in ATH *vs.* MIT pair. Heatmap analysis of the DEGs in plaque *vs.* PBMCs pair revealed the distinct expression pattern between plaque and PBMCs samples (Fig. 2C), while several ATH and MIT samples were not clearly separated (Additional file 1: Fig. S2A). We then analyzed the functions of DEGs. The down DEGs in plaque samples were mainly enriched in immune response-related terms, including innate immune response, T cell receptor signaling pathway, and immune response (Fig. 2D). The up DEGs in plaque samples were mainly enriched in ECM-related terms, including collagen catabolic process, extracellular matrix disassembly, cell adhesion, and angiogenesis (Fig. 2E). KEGG enrichment analysis for up DEGs and down DEGs also showed similar results (Figure S2B-C). Meanwhile, functions of DEGs from ATH vs. MIT pair showed a reverse pattern with immune response terms enriched in up DEGs (Fig. 2F) and ECM-related terms enriched in down DEGs (Fig. 2G). We observed that focal adhesion and ECM-receptor interaction pathways were also enriched in DMGs (Additional file 1: Fig. S1A). We then analyzed gene expression pattern from these two pathways in Additional file 1: Fig. S1A, and found half of them were consistently elevated in plaque samples and the other half of the genes also showed higher expression in several plaque samples (Additional file 1: Fig. S2D–E), suggesting that DNA methylation alternation could influence the expression levels of genes.

### Analysis of the dynamics of immune cell population in atherosclerotic carotid plaques and PBMCs

**Fig. 3:**
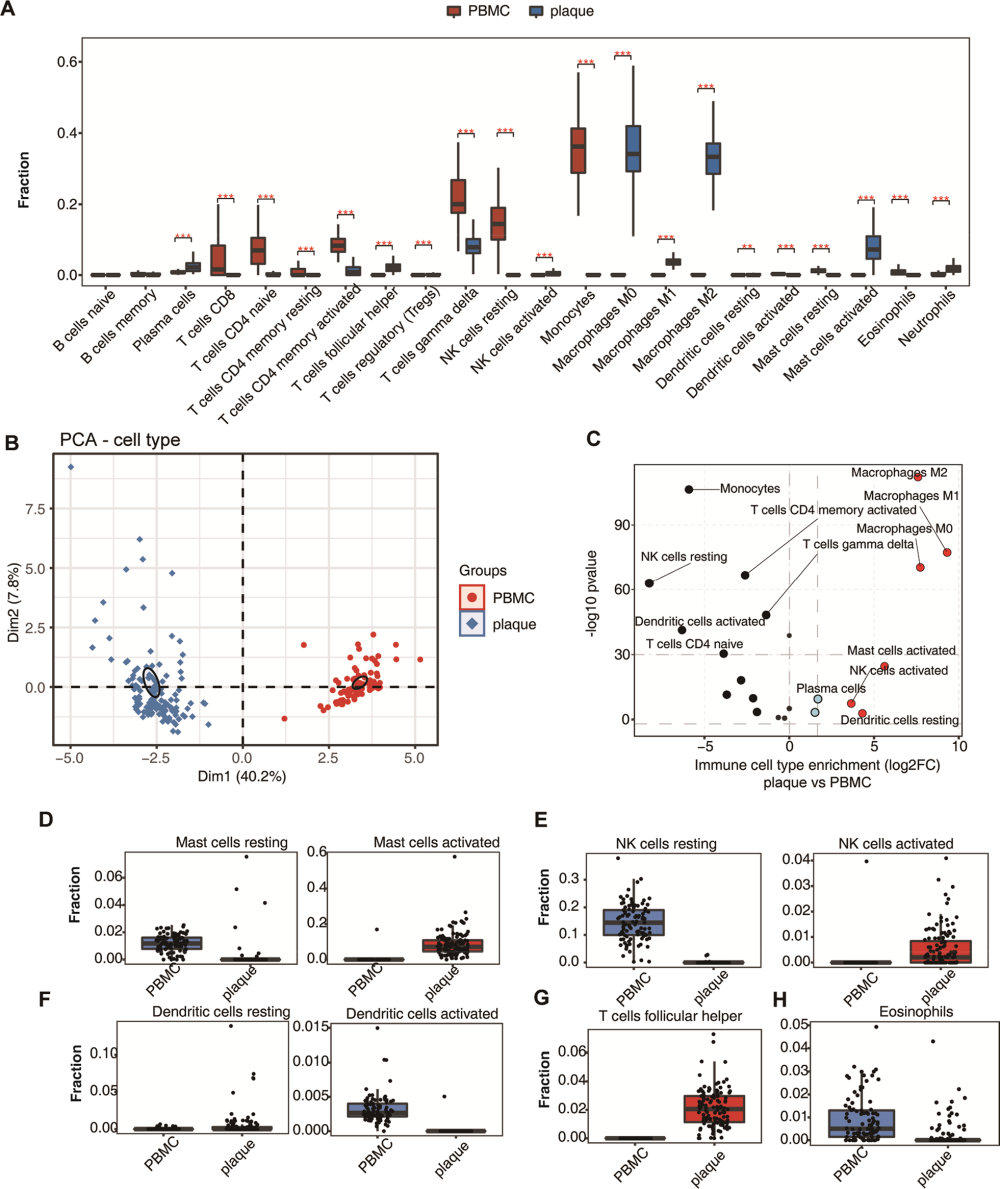
The dynamics of cell population in atherosclerotic carotid plaques and PBMCs. (**A**) Bar plot showing the fractions of immune cell types estimated by CIBERSORT in plaque and PBMCs groups. ***p value < 0.001, unpaired *t*-test. (**B**) Principal component analysis (PCA) of samples based on the proportion of different cell types. The samples were grouped by disease state. The ellipse for each group is the confidence ellipse. (**C**) Dot plot showing the fraction changes (X-axis, log_2_ fold changes, log_2_FC) and statistical *p*-values (Y-axis, -log_10_*P*-value) of immune cell types between plaque and PBMCs. (**D**) Scatter box plots showing the proportion of resting and activated mast cells in atherosclerotic carotid plaques and PBMCs. (**E**) The same with D but for the resting and activated NK cells. (**F**) The same with D but for the resting and activated DC cells. (**G**) The same with D but for the T follicular helper cells. (**H**) The same with D but for the Eosinophils cells

Clinical samples often show more diversity than cell line samples because of their heterogeneity with multiple cell types. It is important to decipher the main cell types, especially immune cells, and the relative percentage change of these cells in atherosclerotic carotid plaque samples. We used CIBERSORT software [31] to estimate the relative fractions of immune cells using expression profiles in plaque tissues and PBMCs. Except for uncharacterized cells, a total of 22 immune-cell types were identified. Fraction analysis of each cell type showed a dramatic difference between plaque and PBMC samples (Fig. 3A). Macrophages and mast activated cells were dominant in plaque samples, while immune T cells, monocyte, and resting NK cells contributed a high fraction in PBMC samples (Fig. 3A). We also observed that gamma delta T cells were with high fraction (> 0.1) in both plaque and PBMC samples (Fig. 3A). We then performed PCA analysis to estimate the immune cell fraction influence on sample distribution. The result showed that immune cell fractions had the ability to identify plaque samples from PBMC samples (Fig. 3B), validating the distinct cell type differences in these two groups. We estimated the relative fraction difference of each cell type by calculating log_2_ fold change (log_2_FC) and *p*-value in plaque samples *vs.* PBMCs. Although with a low fraction, resting NK cells showed the highest absolute fold change among PBMCs enriched cell types (Fig. 3C). Various CD4 + T cell types, including native, activated, and resting, as well as activated dendritic cells, and monocytes were significantly enriched in PBMC samples (Fig. 3C), which was consistent with the natural composition of PBMCs [36]. Three types of macrophages, including M0, M1 and M2, were dominantly enriched in plaques with highly significant *p* values and fold changes. Other cell types also showed significant differences between plaque and. PBMCs (Fig. 3C). We then showed the detailed fractions of each immune cell type that were enriched in atherosclerotic plaque samples or PBMCs. Macrophages and several T cell types showed a high fraction in plaque and PBMC samples, respectively (Additional file 1: Fig. S3A–B). Despite the cells with fractions of more than 0.1, we observed that the resting and activated mast cells were specifically enriched in PBMC and plaque samples, respectively (Fig. 3D). This fraction shift between PBMC and plaque samples for the same cell type with different cellular states was also observed for natural killer (NC) cells, showing elevated fraction of activated state and decreased fraction of resting state in plaque samples (Fig. 3E). For dendritic cells (DCs), only activated DCs were enriched in PBMCs, and resting DCs showed no differences between PBMC and plaque samples (Fig. 3F). We also observed that T follicular helper cells (Tfh cells) showed higher fraction in plaques (Fig. 3G), while other T cells were enriched in PBMC samples (Additional file 1: Fig. S3B). Eosinophils cells, with immunomodulatory functions and homeostasis promotion [37], showed higher fraction in PBMCs (Fig. 3H). Other cell types, including naïve and memory B cells, neutrophils and plasma cells, showed very low fractions and small differences between plaque and PBMC samples (Additional file 1: Fig. S3C). These results demonstrated that the immune cell fractions were greatly affected in atherosclerotic carotid plaques, suggesting that these cell types enriched in carotid plaques might modulate the progression of plaques.

### Integrated analysis of deregulated DNA methylation, gene expression and immune cell population

**Fig. 4:**
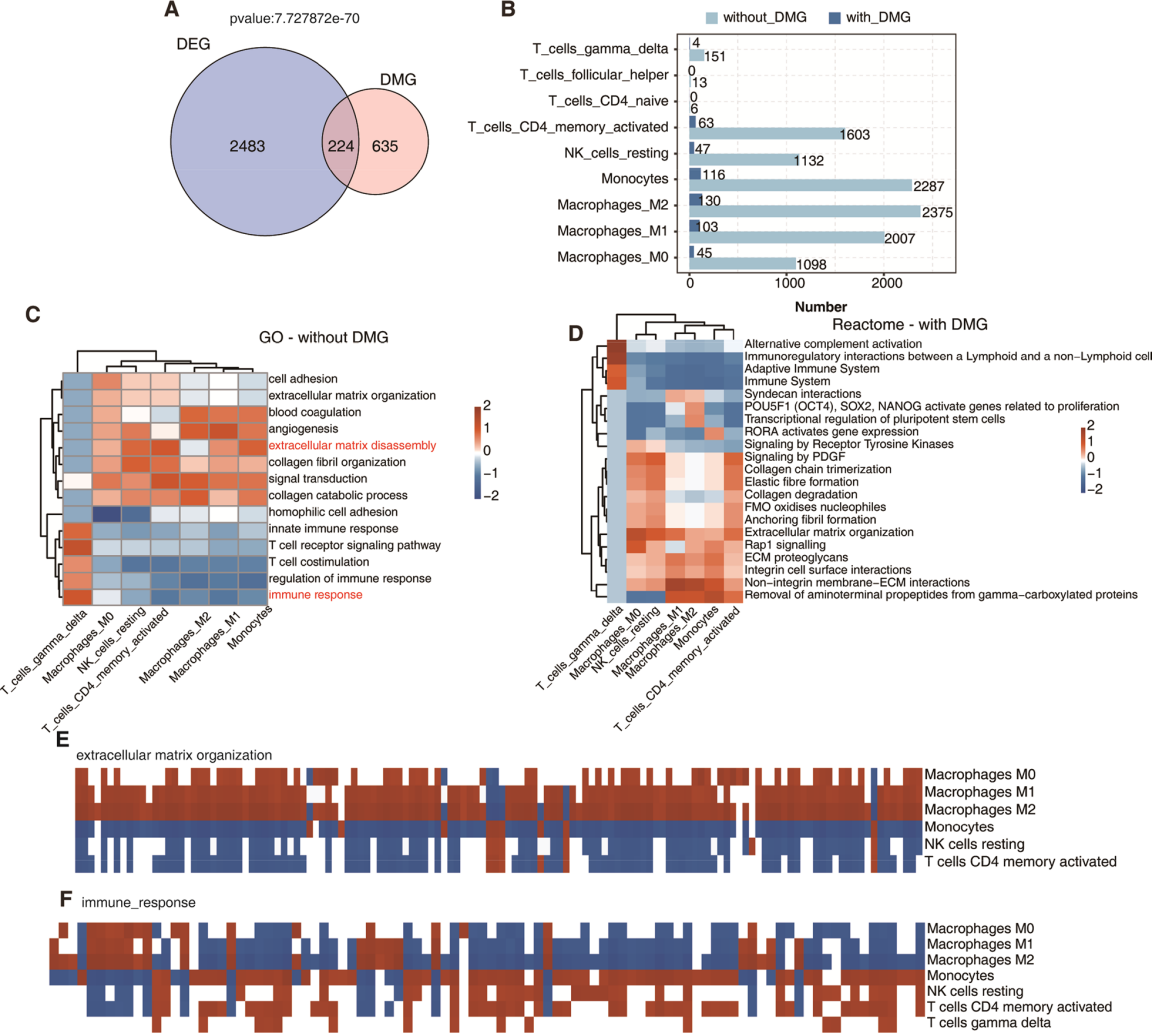
Integrated analysis of deregulated DNA methylation, gene expression and immune cell population. (**A**) Venn diagram showing the overlap between DEGs and DMGs. (**B**) Bar plot showing cell type ratio and the number of co-expressed DEGs with DMG or without DMG. (**C**) Hierarchical clustering heatmap showing the correlation between enriched GO terms (biological process) and cell types for co-expressed DEGs without DMG. (**D**) Hierarchical clustering heatmap showing the correlation between enriched Reactome terms and cell types for co-expressed DEGs with DMG. (**E**) Heatmap showing the correlation pattern between expression levels of genes from extracellular matrix organization and the fractions of related immune cell types. Red color indicates positive correlation, and blue color indicates negative correlation. (**F**) The same with E but for the expression levels of genes from immune response and the fractions of related cell types

To figure out how DNA methylation influences gene expression, we made an integration analysis between DEGs and DMGs using the two published datasets. The results showed that 224 DEGs in atherosclerotic carotid plaque samples also had DNA methylation change at their promoter region, accounting for 26% of total DMGs (Fig. 4A, *p* value = 7.73e-70, hypergeometric test). Functional analysis of the 224 overlapped genes revealed they were highly associated with ECM organization, cell adhesion, and focal adhesion-related pathways (Additional file 1: Fig. S4A-B), suggesting ECM was dysregulated in plaque by modulating the DNA methylation levels of related genes. We classified these DEGs into immune-cell types by their prior classification in immunedeconv package [30], and then conducted a correlation analysis of the DEG (with or without DMGs) expression level and the cell population percentages from plaque and PBMC samples. The cell type populations and their co-expressed DEG numbers were shown in Fig. 4B. We found that most of the non-methylated DEGs were highly correlated with the macrophages, monocytes, and CD4 + memory activated T cells (Fig. 4B). Resting NK cells were also correlated with 1132 non-methylated DEGs and 47 methylated DEGs. Meanwhile, we found that gamma-delta T cells were correlated with 151 non-methylated DEGs and 4 methylated DEGs, ranking second among T cells (Fig. 4B). We then constructed the relationship between cell types and the functions of their correlated DEGs with methylated or without methylated changes after classifying them into immune cell types. For DEGs without DNA methylation change, heatmap plot of the enriched GO terms showed immune response and T cell stimulation terms were specifically and positively correlated with gamma-delta T cells (Fig. 4C). While ECM-related terms had a positive correlation with macrophages, resting NK cells, CD4 + memory-activated T cells and monocytes (Fig. 4C). KEGG analysis of DEGs without DNA methylation change showed that these highly correlated cell types had similar enriched pathways (Additional file 1: Fig. S4C). Reactome and KEGG pathway analysis of DEGs with DNA methylation change showed that gamma-delta T cells were also positively correlated with immune system-related pathways (Fig. 4D). Other immune cell types, including macrophages, resting NK cell, monocytes, and activated CD4 memory T cells, were positively correlated in ECM-related pathways (Fig. 4D and Additional file 1: Fig. S4D). We then performed correlation analysis between cell infiltration and immune response or ECM organization gene expression in the datasets (absolute correlation coefficient > 0.8 and *p* value < 0.01, Additional file 5: Table S4). Strikingly, most of the genes from ECM organization were positively correlated with three types of macrophages, and negatively correlated with monocytes, resting NK cells, and activated CD4 memory T cells (Fig. 4E). We also checked genes from focal adhesion pathway, and found they showed a similar correlation pattern with genes from ECM organization pathway (Additional file 1: Fig. S4E). Meanwhile, genes from immune response pathway showed a contrary correlation pattern with immune cell types compared with genes from ECM organization (Fig. 4F).

### Verification of genes deregulated at both expression and DNA methylation levels in atherosclerotic clinical samples

**Fig. 5:**
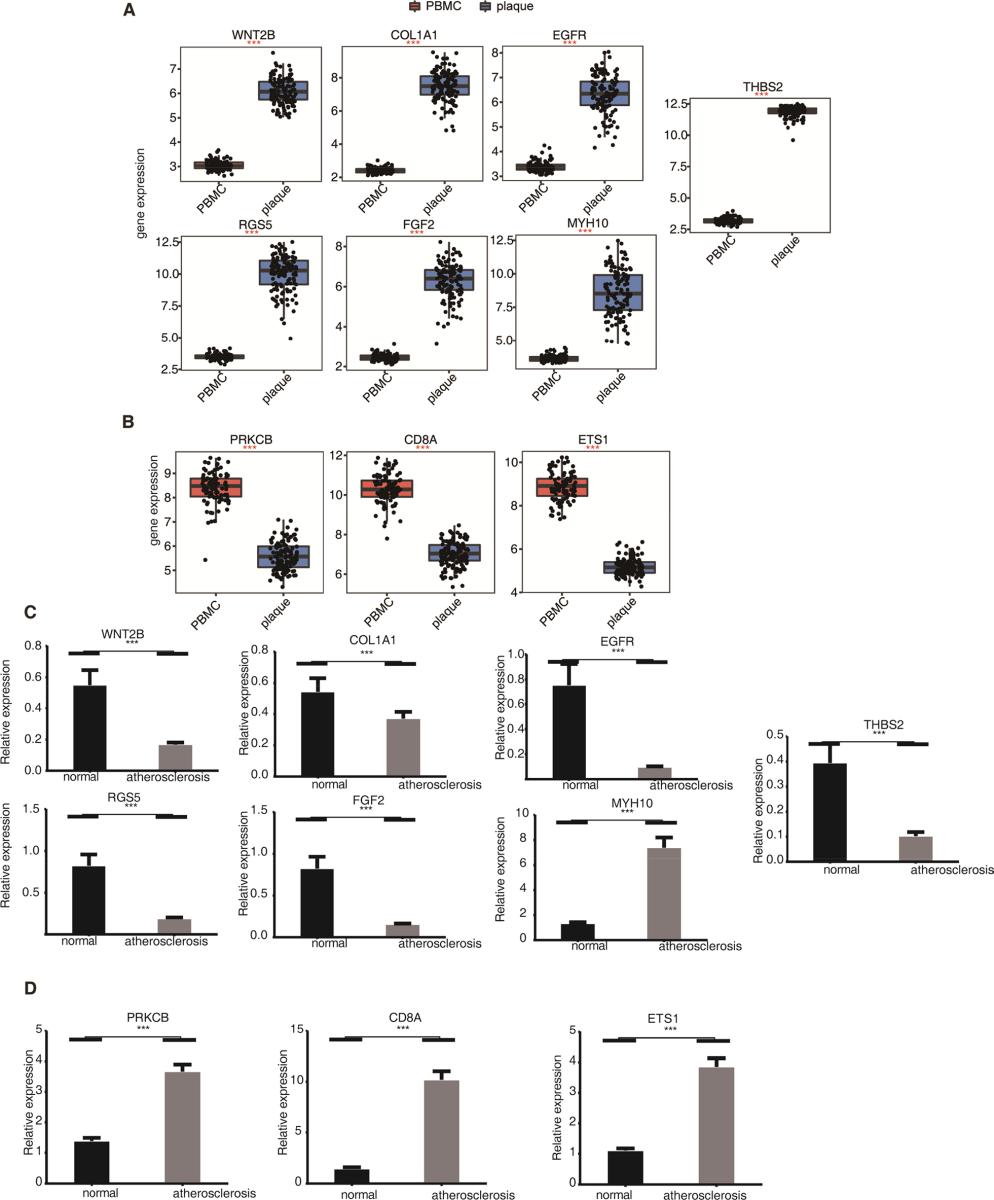
Verification of genes deregulated at both expression and DNA methylation levels in atherosclerotic clinical samples. (**A**). Box plot showing the expression levels of 7 genes that were down-regulated in atherosclerotic PBMCs compared with those in carotid plaques samples. ***p value < 0.001, unpaired *t*-test. (**B**) The same with A but for three up-regulated genes in atherosclerotic PBMCs. (**C**) Bar plot showing the RT-qPCR results of 7 genes from (**A**) in PBMCs samples from normal individuals and atherosclerotic patients. ***p value < 0.001, unpaired *t*-test. (**D**) The same with C but for three genes from (**B**)

To further validate the relationship between cell composition and gene expression in atherosclerosis, we conducted an RT-qPCR experiment for several genes. We selected ten genes that were both differentially methylated and differentially expressed in atherosclerotic carotid plaques, including *COL1A1*, *THBS2*, *RGS5*, *PRKCB*, *MYH10*, *FGF2*, *WNT2B*, *ETS1*, *CD8A*, and *EGFR*, to explore their expression changes in atherosclerosis patients. Due to limited resources and time, plaque samples could not be obtained. We extracted PBMCs from 15 patients and control samples (See methods for detailed information). Immune cell correlation analysis revealed they were associated with macrophages, monocytes, resting NK cells, activated CD4 memory T cells, and gamma delta T cells (Additional file 6: Table S5). Functional annotation revealed these genes were associated with ECM disassembly/organization (COL1A1, FGF2), cell adhesion (THBS2, MYH10), signal transduction (RGS5), blood coagulation (PRKCB), cell fate commitment (WNT2B), and immune response (ETS1). Box plot analysis of the 10 genes showed that 7 and 3 genes were down-regulated and up-regulated in atherosclerotic PBMCs, respectively (Fig. 5A–B). In this study, PBMCs were extracted from 15 atherosclerosis patients and 15 normal control individuals. Using the RT-qPCR method, we found that all these 10 genes showed significant differences between atherosclerosis patients and normal controls (Fig. 5C–D). COL1A1, THBS2, RGS5, FGF2, WNT2B, and EGFR were down-regulated in atherosclerotic PBMCs, while PRKCB, MYH10, ETS1, and CD8A were up-regulated in atherosclerotic PBMCs (Fig. 5B–C). These results revealed that, except for MYH10, the changing tendency of the other nine genes was completely consistent between normal PBMCs and plaque samples, compared with that between the PBMC samples from atherosclerotic patients. The gene expression array experiment was performed using carotid plaques and PBMCs from atherosclerotic patients, and the RT-qPCR experiment was performed using PBMCs from atherosclerotic patients and control individuals. We found that these nine genes showed a high expression variation in PBMCs from atherosclerotic patients, suggesting that the differentially expressed genes in PBMC cells from atherosclerotic patients may play important roles in the formation of plaques. Our study highlights the regulatory roles of key genes associated with infiltrating immune cells in atherosclerosis.

## Discussion

At molecular level, the pathogenesis of atherosclerosis is associated with multiple factors. Transcriptional and epigenetic regulation of macrophages is a major driver of atherosclerosis [21]. In this study, we integrated DNA methylation profile and expression profile from atherosclerosis and control individuals to decipher how DNA methylation modulates the progression of atherosclerosis by regulating transcription of genes involved in atherosclerosis, and try to investigate the composition of immune cell types in atherosclerosis. We found that the DNA methylation profile showed a distinct pattern among normal aortic, atherosclerotic aorta, and atherosclerotic carotid plaque samples. The DMGs and DEGs interaction analysis demonstrated that hundreds of genes had expression changes which may be caused by DNA methylation regulation at the promoter region, and that these genes were tightly associated with atherosclerosis. We explored the immune-cell fraction changes in atherosclerotic carotid plaque samples and PBMCs and found that plaque samples showed distinct immune cellular fraction distribution. Several activating immune cells, including NK cells and mast cells, were specifically enriched in plaque cells. Further analysis revealed that these specifically enriched cell types were highly correlated with immune response and ECM organization-related pathways associated with the formation and progression of plaques [38]. Taken together, our results highlighted the important roles of DNA methylation on gene expression changes and proved that specific immune cell types play potential functions during atherosclerosis development and progression.

DNA methylation alteration is one of the most important epigenetic regulation manners. Several studies have demonstrated the global DNA methylation changes between atherosclerosis and normal individuals [26, 39]. A recent review paper suggested that targeting the epigenetic landscape of plaque macrophages can be a powerful therapeutic tool to modulate pro-atherogenic phenotypes and reduce the rate of plaque formation [21]. Correlation analysis between DNA methylation drift and histological grade showed that hypermethylation was associated with lesion progression [40]. CD14+ blood monocyte transcriptome and epigenome signatures suggest that *ARID5B* expression, possibly regulated by an epigenetically controlled enhancer, promotes atherosclerosis by dysregulating immunometabolism towards a chronic inflammatory phenotype [41]. DMRs in the promoter region of BRCA1 and CRISP2 were consistently associated with subclinical atherosclerosis measures, suggesting their potential blood surrogate markers for early risk stratification [42]. We found that the global DNA methylation profile showed a distinct pattern between atherosclerotic lesions and donor-matched normal samples. Adhesion junction and blood coagulation were the most enriched pathways in DMGs. Cellular adhesion molecules are the dominant members to recruit inflammatory cells to vascular endothelium [43]. While blood coagulation is an essential determinant of the risk of atherothrombotic complications [44]. DNA methylation changes around the promoter region of these genes could trigger following transcriptional and post-transcriptional alternations. These results demonstrated that DNA methylation could regulate atherosclerosis by modulating the status of CpG islands of associated genes.

The immune cell infiltration is a prominent feature of the adipose tissue inflammation, which leads to vascular remodeling and contributes to vascular disease, atherosclerosis, and plaque instability [45]. Several studies have shown that immune-cell types have different DNA methylation patterns in many diseases, including multiple sclerosis [46], type 1 diabetes [47], and metastatic melanoma [48]. By classifying the expressed genes into immune cell types, we found that the fraction of immune cells showed significant changes between plaque tissues and PBMCs. Higher fractions of macrophages, including M0, M1 and M2, were observed in plaque tissues. It has been demonstrated that macrophage phenotypes dysregulation plays a major driver in atherosclerosis, including the transcriptional and epigenetic heterogeneity [21]. Our data suggests that macrophages are dysregulated in atherosclerotic plaque tissues, and that they participate in the inflammatory progression of atherosclerosis by accumulating their fraction. One protein-gene-associated multi-omics model between low- and high-risk lesion segments revealed that it was corrected with Arg1^+^ macrophage content and αSMA^−^PDGFRα^+^ fibroblast-like cell content [49]. We also found that M2 macrophage (Arg1^+^) showed a higher fraction in plaque (Fig. 3A), but fibroblast-like cells were not fully identified. Gene set over-representation analysis pointed to a clear cardiovascular disease signature, including extracellular matrix synthesis and organization, and focal adhesion [49], which were also observed in enriched pathways of DEGs. Meanwhile, T follicular helper (Tfh) cells were also specifically enriched in plaque samples. Tfh cells play important roles in many diseases during the decade from their identification [50]. A study has demonstrated their pro-atherogenic roles in a Bcl6 mouse model, and proved their existence in the plasma of human subjects with coronary artery disease [51]. The high fraction of Tfh cells in the plaque from our study revealed their regulatory roles during plaque formation. Other T cells were mostly enriched in PBMC samples of atherosclerosis (Additional file 1: Fig. S3B). We also discovered that activating mast cells and natural killer (NK) cells were enriched in plaque samples, while their resting cells were enriched in PBMC samples. It has been reported that mast cells were accumulated in human atherosclerotic lesions [52] and could promote atherosclerosis by releasing proinflammatory cytokines [53]. NK cells could induce an immune response and participate in the pathogenesis and progression of atherosclerosis [54]. The infiltration of immune cells from blood to vessel is closely associated with the progression and prognosis of atherosclerosis [55]. The state transition of these two cells suggests that they could infiltrate into plaque and be activated by other factors to function, the process of which needs to be uncovered by further studies. Collectively, our study demonstrated that PBMC and plaque tissues have very distinct immune cell fractions and that their population changes are phenotypes of atherosclerosis and associated with complex plaques that may be related to clinical events. Recent studies on single-cell technology also demonstrated the various cell types in plaque samples of AS patients, which extended our understanding of immune cell infiltration during AS development [56, 57]. One shortage of this study is that the identified immune cell fraction is unitary as we did not consider the other cell types due to technical limitation. Thus, it will be very helpful to make a deeper exploration of immune cell variation in plaques using single-cell technology in future studies.

We then integrated the DEGs and DMGs to further identify the expression outcomes of DNA methylation of associated genes. Out of the 859 DMGs, 224 showed significant expression changes in plaque tissues. Functional analysis of these overlapped genes demonstrated that they were highly related to cell adhesion and ECM organization, suggesting that these genes participate in the progression of atherosclerosis by altering the ECM structure of plaque tissues. The connection between immune cell fractions and biological functions was analyzed on the basis of the gene expression data. Several immune cells were found to be specifically associated with extracellular inflammatory-related pathways or immune response-related pathways besides immune- and inflammatory-related pathways. Gamma delta (γδ) T cells were positively correlated with immune response pathways, while macrophages, resting NK cells, monocytes, and activated CD4 memory T cells, were positively correlated in ECM-related pathways. In the multi-ethnic study of atherosclerosis (MESA), γδ T cells are associated with systolic blood pressure [58]. However, it is also reported that γδ T cells do not contribute to the early atherosclerotic plaque development by generating TCRδ knockout ApoE^−/−^ mice [59]. These results indicate that the functions of γδ T cells in atherosclerosis are not fully understood and need to be deciphered with further studies. Among the immune cell types correlating with the ECM-related pathways, macrophages were enriched in plaques, while resting NK cells, monocytes, and activated CD4 memory T cells were enriched in PBMC samples (Fig. 3 and Additional file 1: Fig. S3). The association between these immune cells and atherosclerosis has been discussed. These cells have positive correlation with genes involved in ECM, which is composed of various macromolecules and plays important roles during the development of atherosclerotic plaques [60, 61]. Then we selected 10 genes that were correlated with immune cell types shown in Fig. 4C–D to investigate their expression in PBMC samples. These genes contained WNT2B, COL1A1, EGFR, CD8A, and ETS1 and have strong biological implications that can be linked with WNT and EGFR signaling (WNT2B and EGFR), collagen production (COL1A1 and ETS1) and immune cells (CD8A). The validation of these genes suggests that their expressions were highly regulated in PBMCs between AS patients and normal samples. It is very interesting that they all had significant expression changes in atherosclerotic PBMC samples versus normal samples. When compared with atherosclerotic PBMC samples, they showed a consistent expression change between normal PBMC and plaque samples. One explanation of this phenomenon is that immune cells expressing these genes in the blood infiltrate into the vessel wall and trigger the formation of plaques, resulting in the reduction of these cells in the atherosclerotic PBMC samples. Several recently published studies have demonstrated the profile of immune cell infiltration and the potential regulatory genes in the progression of atherosclerosis [62–64], suggesting that these genes identified in this study may also play important roles in immune cell infiltration and plaque development. Further investigations into the molecular mechanisms of these genes in atherosclerosis could greatly help us understand the pathogenesis of plaque formation.

In summary, a comprehensive analysis was made to explore the DNA methylation and its regulated transcriptome profile changes in atherosclerotic plaques. The high correlation between DMGs and DEGs revealed their potential regulatory roles and functions in immune cell infiltration. Meanwhile, we also systematically investigated the immune cell alteration in atherosclerotic plaque samples and identified several immune cell types tightly associated with plaque formation and development. Our study highlights the dysregulated methylation and expression levels of key genes associated with infiltrating immune cells in atherosclerosis, extending our understanding of the immune cell infiltration and its potential underlying mechanisms during atherosclerosis pathogenesis or development.

## Supplementary Information

**Additional file 1: Figure S1**. Analysis of the hypermethylated genes previously identified in atherosclerotic aortas and carotid plaques. A. Top 10 most enriched KEGG pathways of differential methylated genes (DMG). B. Top 10 most enriched Reactome pathways of differential methylated genes (DMG). **Figure S2**. Transcriptome analysis of deregulated in atherosclerotic carotid plaques.A. Expression heatmap of all differentially expressed genes between ATH vs. MIT. B-C. Top 10 most enriched KEGG pathways of up-regulated (A) down-regulated (B) genes from differential expressed genes compared atherosclerotic carotid plaque with PBMCs. D. Hierarchical clustering heat map showing the expression level of DNAm changed genes involved in focal adhesion. E. Hierarchical clustering heat map showing the expression level of DNAm changed genes involved in ECM receptor interaction. **Figure S3**. Transcriptome analysis of the dynamics of cell population in atherosclerotic carotid plaques. A. Scatter box plots showing proportion of three macrophage cell types in atherosclerotic carotid plaques and PBMCs. *** p-value < 0.001, unpaired t-test. B. The same with A but for the various T cell types in atherosclerotic carotid plaques and PBMCs. C. The same with A but for the naïve and memory B cells, neutrophils and plasma cells in atherosclerotic carotid plaques and PBMCs. **Figure S4**. Integrated analysis of deregulated DNA methylation, gene expression and immune cell population. A. Top 10 most enriched GO terms (biological process) by overlapped genes of DEG and DMG. B. Top 10 most enriched KEGG pathways by overlapped genes of DEG and DMG. C. Hierarchical clustering heat map showing the correlation between enriched KEGG pathways and cell types for co-expressed DEGs without DMG. D. Hierarchical clustering heat map showing the correlation between enriched KEGG pathways and cell types for co-expressed DEGs with DMG. E. Heat map showing the correlation pattern of gene expression from focal adhesion and related cell types. Red color indicates positive correlation, and blue color indicates negative correlation.

**Additional file 2: Table S1**. Clinical information table of patients.

**Additional file 3: Table S2**. Primer table for RT-qPCR experiment.

**Additional file 4: Table S3**. The detial DNA methylation density of DMGs. All PBMC and plaque samples were shown for each gene.

**Additional file 5: Table S4**. Correlation information between fraction of cell types and expression level of genes.

**Additional file 6: Table S5.** Ten_genes correlated with immune cell types.

## Data Availability

The datasets analyzed during the current study are available in the NCBI GEO repository, including GSE46401, GSE43292, and the BiKE cohort (GSE21545).
