# Supplementary material for: Integrated investigation of DNA methylation, gene expression and immune cell population revealed immune cell infiltration associated with atherosclerotic plaque formation
Source: BMC Med Genomics. 2022 May 9;15:108. doi: 10.1186/s12920-022-01259-z (PMC9082837; doi:10.1186/s12920-022-01259-z)
Supplement: Supplementary file 1 — Additional file 1: Figure S1. Analysis of the hypermethylated genes previously identified in atherosclerotic aortas and carotid plaques. A. Top 10 most enriched KEGG pathways of differential methylated genes (DMG). B. Top 10 most enriched Reactome pathways of differential methylated genes (DMG). Figure S2. Transcriptome analysis of deregulated in atherosclerotic carotid plaques.A. Expression heatmap of all differentially expressed genes between ATH vs. MIT. B-C. Top 10 most enriched KEGG pathways of up-regulated (A) down-regulated (B) genes from differential expressed genes compared atherosclerotic carotid plaque with PBMCs. D. Hierarchical clustering heat map showing the expression level of DNAm changed genes involved in focal adhesion. E. Hierarchical clustering heat map showing the expression level of DNAm changed genes involved in ECM receptor interaction. Figure S3. Transcriptome analysis of the dynamics of cell population in atherosclerotic carotid plaques. A. Scatter box plots showing proportion of three macrophage cell types in atherosclerotic carotid plaques and PBMCs. *** p-value < 0.001, unpaired t-test. B. The same with A but for the various T cell types in atherosclerotic carotid plaques and PBMCs. C. The same with A but for the naïve and memory B cells, neutrophils and plasma cells in atherosclerotic carotid plaques and PBMCs. Figure S4. Integrated analysis of deregulated DNA methylation, gene expression and immune cell population. A. Top 10 most enriched GO terms (biological process) by overlapped genes of DEG and DMG. B. Top 10 most enriched KEGG pathways by overlapped genes of DEG and DMG. C. Hierarchical clustering heat map showing the correlation between enriched KEGG pathways and cell types for co-expressed DEGs without DMG. D. Hierarchical clustering heat map showing the correlation between enriched KEGG pathways and cell types for co-expressed DEGs with DMG. E. Heat map showing the correlation pattern of gene expression from focal adhesion and [file 12920_2022_1259_MOESM1_ESM.docx]

**Supplementary Figures**


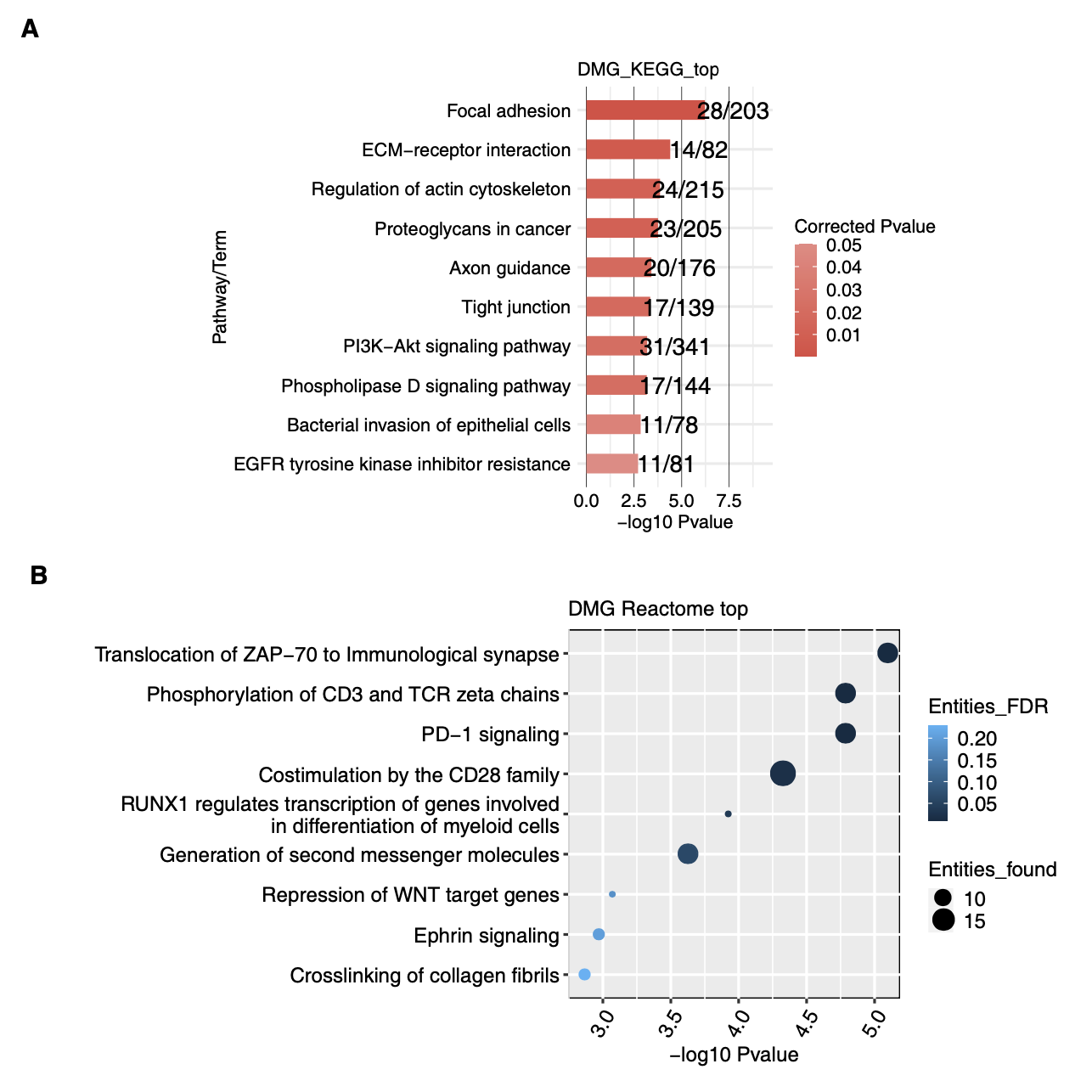


**Figure S1. Analysis of the hypermethylated genes previously identified in atherosclerotic aortas and carotid plaques.**

1. Top 10 most enriched KEGG pathways of differential methylated genes (DMG).
2. Top 10 most enriched Reactome pathways of differential methylated genes (DMG).


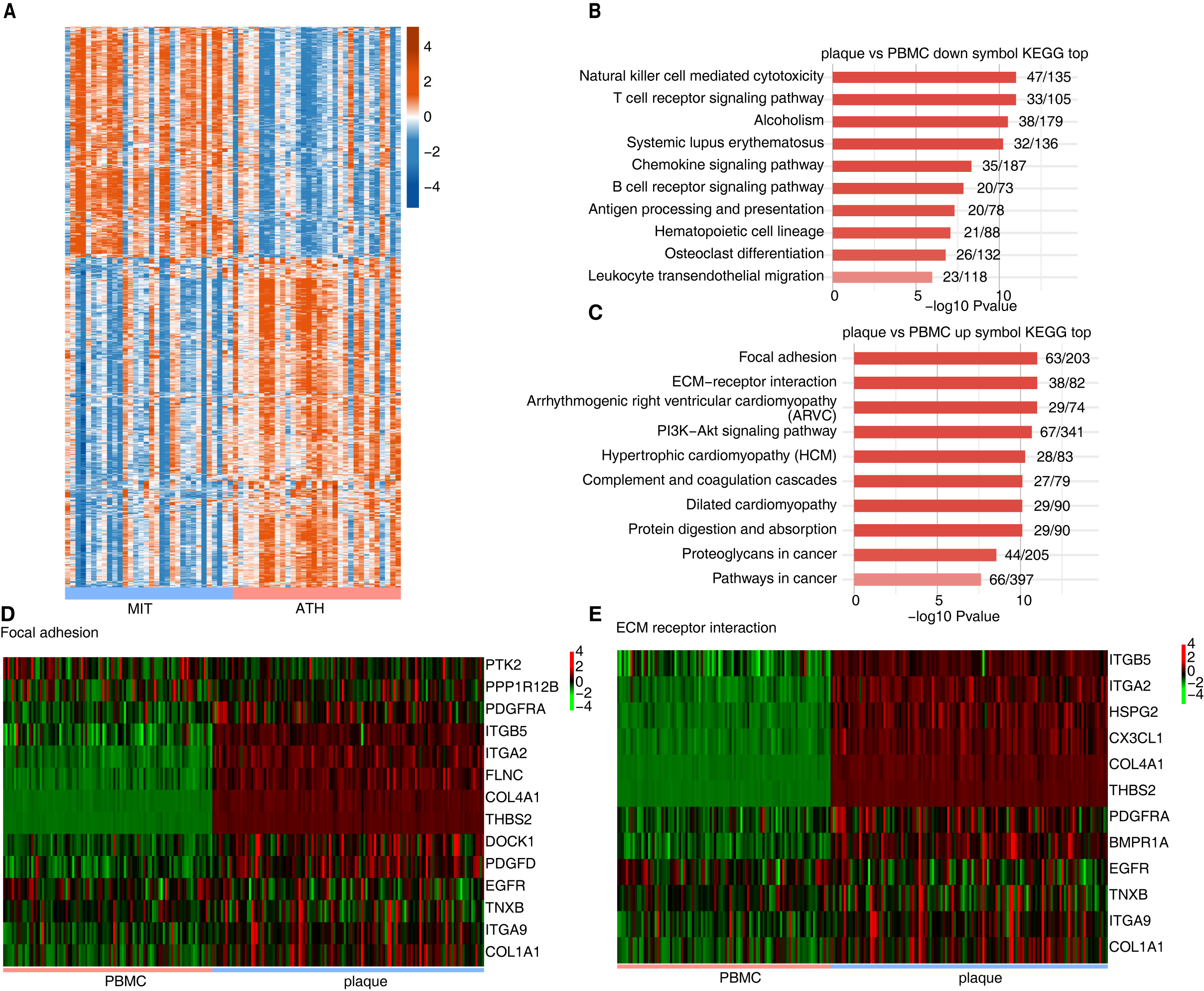


**Figure S2. Transcriptome analysis of deregulated in** **atherosclerotic carotid plaques.**

**A.** Expression heatmap of all differentially expressed genes between ATH *vs.* MIT.

**B-C**. Top 10 most enriched KEGG pathways of up-regulated (A) down-regulated (B) genes from differential expressed genes compared atherosclerotic carotid plaque with PBMCs.

**D.** Hierarchical clustering heat map showing the expression level of DNAm changed genes involved in focal adhesion.

**E.** Hierarchical clustering heat map showing the expression level of DNAm changed genes involved in ECM receptor interaction.


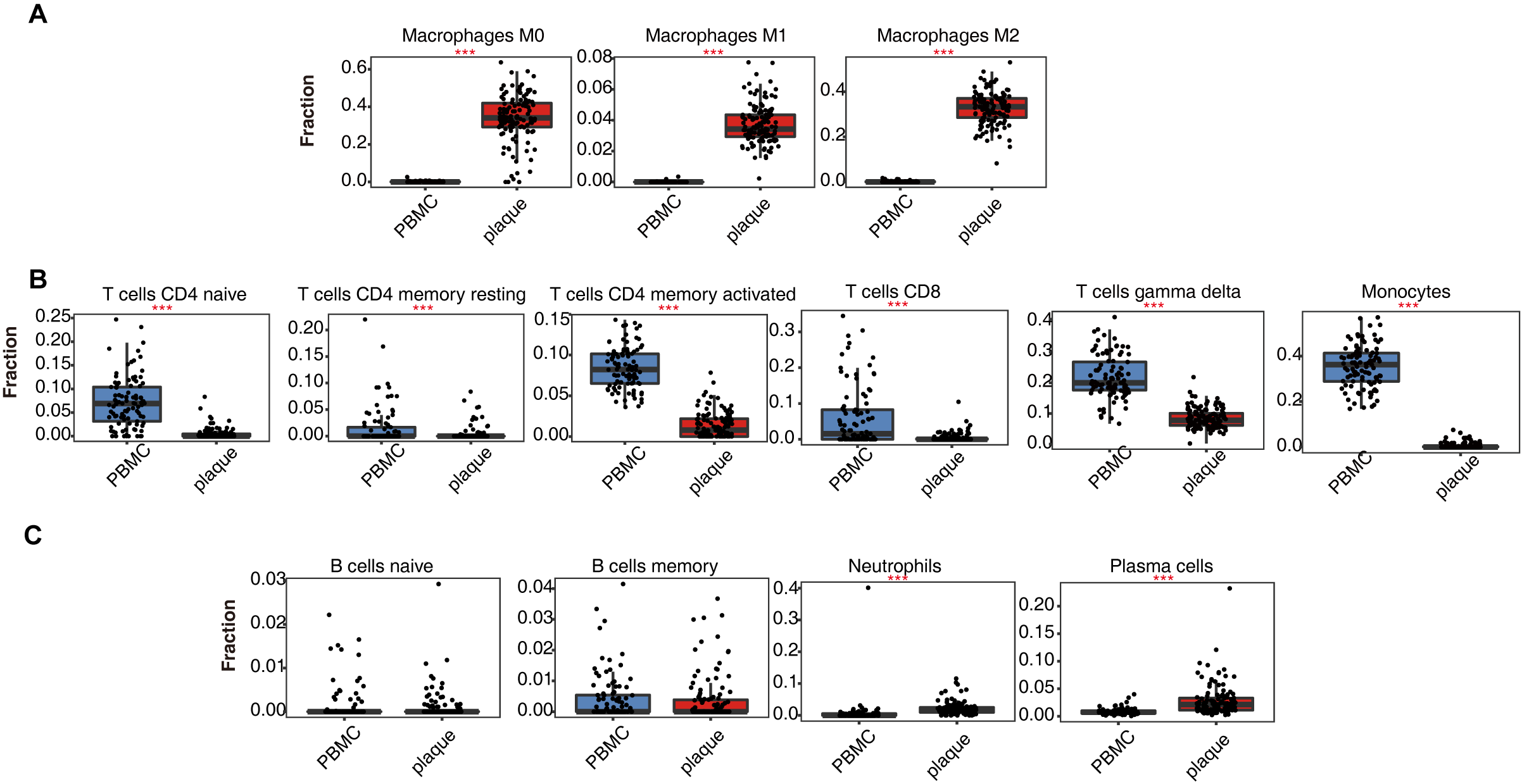


**Figure S3. Transcriptome analysis of the dynamics of cell population in atherosclerotic carotid plaques.**

1. Scatter box plots showing proportion of three macrophage cell types in atherosclerotic carotid plaques and PBMCs. *** *p*-value < 0.001, unpaired *t*-test.
2. The same with A but for the various T cell types in atherosclerotic carotid plaques and PBMCs.
3. The same with A but for the naïve and memory B cells, neutrophils and plasma cells in atherosclerotic carotid plaques and PBMCs.


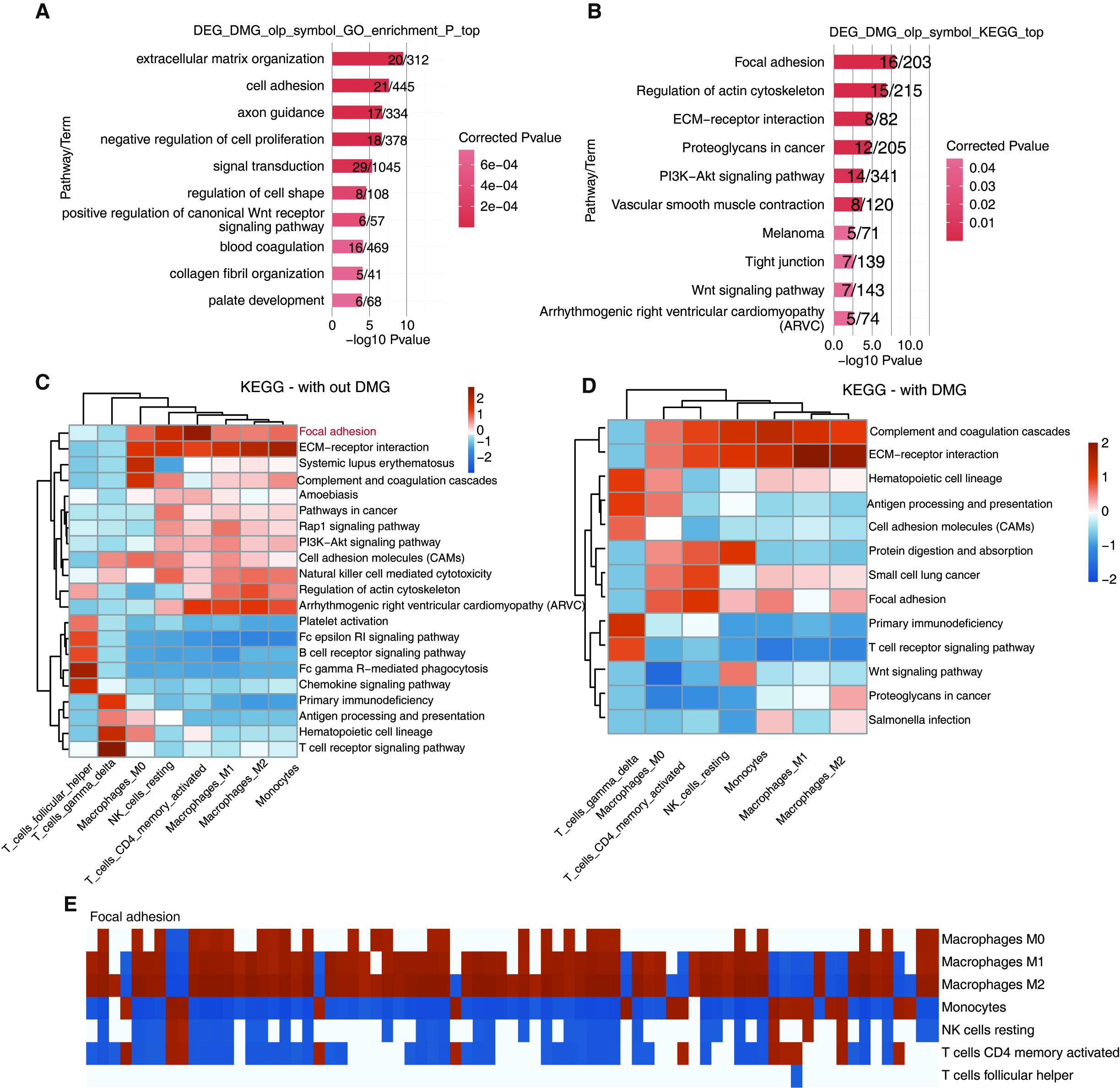


**Figure S4. Integrated analysis of deregulated DNA methylation, gene expression and immune cell population.**

1. Top 10 most enriched GO terms (biological process) by overlapped genes of DEG and DMG.
2. Top 10 most enriched KEGG pathways by overlapped genes of DEG and DMG.
3. Hierarchical clustering heat map showing the correlation between enriched KEGG pathways and cell types for co-expressed DEGs without DMG.
4. Hierarchical clustering heat map showing the correlation between enriched KEGG pathways and cell types for co-expressed DEGs with DMG.
5. Heat map showing the correlation pattern of gene expression from focal adhesion and related cell types. Red color indicates positive correlation, and blue color indicates negative correlation.
