# Supplementary material for: Integrated investigation of DNA methylation, gene expression and immune cell population revealed immune cell infiltration associated with atherosclerotic plaque formation
Source: BMC Med Genomics. 2022 May 9;15:108. doi: 10.1186/s12920-022-01259-z (PMC9082837; doi:10.1186/s12920-022-01259-z)
Supplement: Supplementary file 2 — Additional file 2: Table S1. Clinical information table of patients. [file 12920_2022_1259_MOESM2_ESM.docx]

| Sample ID | Group | Age | Sex | Race | vascular stenosis level by CTA (%) |
| --- | --- | --- | --- | --- | --- |
| C2 | Control | 61 | Female | Han | -- |
| C3 | Control | 45 | Female | Han | -- |
| C6 | Control | 67 | Male | Han | -- |
| C7 | Control | 46 | Female | Han | -- |
| C8 | Control | 45 | Male | Han | -- |
| C9 | Control | 43 | Female | Han | -- |
| C10 | Control | 38 | Female | Han | -- |
| C11 | Control | 44 | Female | Han | -- |
| C12 | Control | 48 | Male | Han | -- |
| C13 | Control | 49 | Male | Han | -- |
| C14 | Control | 42 | Female | Han | -- |
| C15 | Control | 47 | Male | Han | -- |
| C17 | Control | 38 | Male | Han | -- |
| C19 | Control | 43 | Female | Han | -- |
| C20 | Control | 48 | Male | Han | -- |
| AS-1 | Disease | 51 | Male | Han | >50 |
| AS-4 | Disease | 46 | Male | Han | >50 |
| AS-7 | Disease | 51 | Male | Han | >50 |
| AS-8 | Disease | 36 | Male | Han | >50 |
| AS-9 | Disease | 35 | Male | Han | >50 |
| AS-10 | Disease | 39 | Male | Han | >50 |
| AS-11 | Disease | 57 | Female | Han | >50 |
| AS-12 | Disease | 48 | Male | Han | >50 |
| AS-13 | Disease | 50 | Male | Han | >50 |
| AS-14 | Disease | 48 | Male | Han | >50 |
| AS-15 | Disease | 57 | Female | Han | >50 |
| AS-16 | Disease | 59 | Male | Han | >50 |
| AS-17 | Disease | 46 | Male | Han | >50 |
| AS-18 | Disease | 49 | Male | Han | >50 |
| AS-20 | Disease | 53 | Female | Han | >50 |
|  |  |  |  |  |  |

Table S1. Clinical information table of patients
