# Supplementary material for: Integrated investigation of DNA methylation, gene expression and immune cell population revealed immune cell infiltration associated with atherosclerotic plaque formation
Source: BMC Med Genomics. 2022 May 9;15:108. doi: 10.1186/s12920-022-01259-z (PMC9082837; doi:10.1186/s12920-022-01259-z)
Supplement: Supplementary file 3 — Additional file 3: Table S2. Primer table for RT-qPCR experiment. [file 12920_2022_1259_MOESM3_ESM.docx]

| Gene | Forward | Reverse |
| --- | --- | --- |
| COL1A1 | GGATCTCGATCTCGTTGG | AGAACATCACCTACCACTG |
| MYH10 | GTCACTGAATGTCTGTAATACC | ATGCCATCTTCCTGAATGTT |
| WNT2B | AAGGAACAGACGGTTGTG | TTGAGTTGAGAGGCTTGAAT |
| ETS1 | TCTCACCTGACATCCTACC | GACACGACCTAAGTTGAAGA |
| FGF2 | TGTGTTACGGATGAGTGTT | CTCTTAGCAGACATTGGAAG |
| RGS5 | CTGTGATTCTGATTGTGTCTG | ATACTTATCTACTGTCCGAAGG |
| EGFR | CCAGCAGAAGCACTAAGAA | CACCTCTAATACCAGTCATCA |
| CD8A | AGCATGATTCTGAGAACTCT | GCAACAGCCACTACATTAC |
| PRKCB | GCTGGCTTCTCTTATACTAAC | ATACATACGCTTGGCTTGA |
| THBS2 | GTAGGACAAGAGGAGAGTTC | GATTAGAAGACAACAGGCATAG |
| GAPDH | GGTCGGAGTCAACGGATTTG | GGAAGATGGTGATGGGATTTC |

**Table S2. Primer table for RT-qPCR experiment.**
